# Single-operator peroral pancreatoscopy in the preoperative diagnostics of suspected main duct intraductal papillary mucinous neoplasms: efficacy and novel insights on complications

**DOI:** 10.1007/s00464-022-09156-3

**Published:** 2022-03-11

**Authors:** Sini Vehviläinen, Niklas Fagerström, Roberto Valente, Hanna Seppänen, Marianne Udd, Outi Lindström, Harri Mustonen, Fredrik Swahn, Urban Arnelo, Leena Kylänpää

**Affiliations:** 1 Department of Gastrointestinal Surgery, Helsinki University Hospital, Helsinki, Finland; 2 Translational Cancer Medicine Research Program, University of Helsinki, Helsinki, Finland; 3 Department of Upper GI Diseases, Karolinska University Hospital, Stockholm, Sweden; 4 Clinical Science, Intervention and Technology (CLINTEC), Karolinska Institute, Stockholm, Sweden; 5 Department of Surgery, Skåne University Hospital, Lund, Sweden; 6 Department of Surgical and Perioperative Sciences, Surgery, Umeå University, 90185 Umeå, Sweden

**Keywords:** IPMN, Single-operator pancreatoscopy, Post-ERCP pancreatitis, Pancreatitis

## Abstract

**Background:**

Distinguishing intraductal papillary mucinous neoplasms (IPMNs) from other pancreatic cystic lesions is essential since IPMNs carry the risk of becoming malignant. Differentiating the main pancreatic duct involving IPMNs (MD-IPMNs) through conventional imaging is deficient. Single-operator peroral pancreatoscopy (SOPP) represents a promising method offering additional information on suspected lesions in the pancreatic main duct (MD). We aimed to determine the role of SOPP in the preoperative diagnostics of suspected MD-IPMNs and identify factors contributing to SOPP-related complications.

**Materials and Methods:**

In this primarily retrospective study, SOPPs were performed at three high-volume centers on suspected MD-IPMNs. Primary outcome was the clinical impact of SOPP to subsequent patient care. Additionally, we documented post-SOPP complications and analyzed several assumed patient- and procedure-related risk factors.

**Results:**

One hundred and one (101) SOPPs were performed. Subsequent clinical management was affected due to the findings in 86 (85%) cases. Surgery was planned for 29 (29%) patients. A condition other than IPMN explaining MD dilatation was found in 28 (28%) cases. In 35 (35%) cases, follow-up with MRI was continued. Post-SOPP pancreatitis occurred in 20 (20%) patients and one of them was fatal. A decrease in odds of post-SOPP pancreatitis was seen as the MD diameter increases (OR 0.714 for 1.0 mm increase in MD diameter, CI 95% 0.514–0.993, *p* = 0.045). Furthermore, a correlation between lower MD diameter values and higher severity post-SOPP pancreatitis was seen (*T*_JT_ = 599, SE = 116.6, *z* = − 2.31; *p* = 0.020). History of pancreatitis after endoscopic retrograde cholangiopancreatography was a confirmed risk factor for post-SOPP pancreatitis. Conclusions between complications and other risk factors could not be drawn.

**Conclusion:**

SOPP aids clinical decision-making in suspected MD-IPMNs. Risk for post-SOPP pancreatitis is not negligible compared to non-invasive imaging methods. The risk for pancreatitis decreases as the diameter of the MD increases.

Pancreatic cystic lesions are increasingly detected as the frequency of abdominal imaging increases [1], and the prevalence of such lesions correlates strongly with aging [2]. The clinical challenge concerning pancreatic cysts lies in differentiating malignant, premalignant, and benign subtypes [3–6]. Among cystic pancreatic lesions, intraductal papillary mucinous neoplasms (IPMNs) are a common entity [7]. IPMNs harbor the potential of becoming malignant [8]. The clinical management of IPMNs relies on consensus and/or evidence-based guidelines [9, 10]. Worrisome features favoring surgery include main pancreatic duct (MD) dilatation, the presence of contrast uptaking mural nodules, a cyst size exceeding 40 mm, symptoms (e.g., new-onset diabetes mellitus or acute pancreatitis), and cyst growth exceeding 5 mm/year during follow-up [5, 6, 9–12]. In the absence of worrisome features, close surveillance has been suggested [13]. Correctly timed pancreatic resection for IPMN patients improves life expectancy [14]. However, patients with IPMN should not be surgically overtreated, since pancreatic resections also bears its considerable risk of postoperative short- and long-term complications [15, 16].

Distinguishing IPMNs from other pancreatic cystic lesions through magnetic resonance imaging (MRI) or computed tomography (CT) scans can be challenging even if a multidisciplinary team (MDT) conference is consulted [17]. Specifically, confirming MD involvement in IPMNs remains problematic [3, 18]. In addition, side branch subtypes of IPMN (SB-IPMN) also carry a long-term risk of progressing into cancer [19, 20]. Endoscopic ultrasound (EUS) (for morphology: sensitivity 56–78%, specificity 45–67%) in combination with fine-needle aspiration (FNA) (for cytology: sensitivity 28–78%, specificity 83–100%) can be used in addition to improve the diagnostic accuracy in identifying IPMNs from other types of cysts [10, 21].

Although “fish-eye” papillae and oozing of mucus are visualized during duodenoscopy point towards IPMN, these signs are not seen consistently in all patients with MD-IPMN [22]. Endoscopic retrograde cholangiopancreatography (ERCP) is not recommended due to the risk of adverse events, alongside a lower sensitivity and specificity in identifying pancreatic cystic lesions compared to conventional imaging and EUS [10]. Single-operator peroral pancreatoscopy (SOPP), however, has shown promising results by adding visual inspection of the MD and the possibility of gathering visually guided biopsies from suspected areas [22–28].

Here, we aimed to establish the role of SOPP in the preoperative diagnostics of suspected MD-IPMNs. Secondarily, we aimed to identify factors contributing to SOPP-related adverse events.

## Material and methods

In this multicenter cohort study, patients were collected from three high-volume endoscopic centers in Scandinavia. Although a few patients were prospectively included, this study is in essence retrospective. The study population consisted of all patients who underwent SOPP due to MD dilatation and suspected MD-IPMN or mixed-type IPMN (MT-IPMN) at Helsinki University Hospital (HUH), between 2012 and 2019, at Karolinska University Hospital (KUH), between 2015 and 2019, and at Skåne University Hospital (SUS), between 2017 and 2019. Between 2017 and 2019, seven additional patients were recruited prospectively at HUH, all of whom granted their written consent before the procedure. All patient data from KUH and SUS were collected retrospectively. This study received ethical approval from University of Helsinki (document number HUS/428/2017) and Etikprövningsmyndigheten (document number 2021:03989). This study is registered at ClinicalTrials.gov (Identifier: NCT03062124).

All patients were discussed at an MDT conference prior to SOPP. The patients had undergone MRI, CT, or both prior to the MDT conference. In all cases, imaging findings demonstrated cystic appearance of the pancreas and MD dilatation (≥ 5 mm) with the possibility of MD/MT-IPMN. However, consensus on further clinical management could not be reached during MDT conferences based on imaging findings, and further investigations were needed.

The following indications for SOPP were identified: (1) equal possibility for diagnosis other than MD/MT-IPMN that explains the cystic appearance of the pancreas and MD dilatation. Alternative diagnoses such as SB-IPMN, chronic pancreatitis, stricture of the MD, or pancreatic cystadenomas were suspected. A MDT conference recommended SOPP for excluding pathologies other than MD/MT-IPMN before planning surgery. (2) MD/MT-IPMN was highly likely, but the complication risk for pancreatic surgery was increased due to comorbidities such as cardiovascular diseases, prior liver transplant, and abnormal blood coagulation. Some patients were indecisive about surgery. A MDT conference recommended SOPP to gain visual confirmation of MD/MT-IPMN and biopsies on target lesions. Surgery would be reconsidered, if the specimens retrieved provided evidence on high-grade dysplasia (HGD) or malignancy. (3) Pancreatic surgery was indicated based on imaging findings. Due to comorbidities the patient was not eligible for total pancreatectomy. However, a pancreaticoduodenectomy or a distal pancreatic resection would be possible. A MDT conference recommended SOPP to evaluate extension of IPMN lesions in MD in order to make a decision on surgery.

The equipment used for SOPPs primarily relied on SpyGlass DS Direct Visualization System (SpyGlass DS; Boston Scientific, USA). However, 15 (15%) procedures in the early years of this study were performed using older SpyGlass (Boston Scientific, USA) equipment. Indefinite tumorous alterations were examined and biopsied (SpyBite™) as an indicative of MD-IPMN, and the extension of these lesions in the MD (tail, body, neck or head) was noted. Irrigation fluid and brush cytology samples from the MD were collected whenever suitable. All procedures were performed by experienced specialists.

As a key outcome, we determined how often the visual appearance of MD and/or MD irrigation fluid samples and biopsies taken had any impact on further clinical management. Additionally, we collected data from medical charts regarding adverse events (Cotton consensus criteria) [29], diameter of the MD (MRI scan), history of pancreatitis or prior post-ERCP pancreatitis, prophylactic usage of nonsteroidal anti-inflammatory drugs (NSAIDs), and pancreatic stents. Procedure-related data, such as time consumption, timing of the pancreatic sphincterotomy (PS), papillary, or MD orifice balloon dilatation, and occurrence of parallel therapeutic interventions performed [e.g., radiofrequency ablation (RFA) and electrohydraulic lithotripsy (EHL)] were collected. Data on general patient characteristics such as gender, age, the American Society of Anesthesiologists’ physical status classification (ASA), serum carbohydrate antigen 19–9 levels (s-Ca19–9), and body mass index (BMI) were registered.

### Statistical analysis

Data are reported as the number of cases and as percentages for categorical variables. For continuous variables with a normal distribution, mean and standard deviation (SD) are presented. For continuous variables with non-normal distribution, median and range are presented. Fisher’s exact test was used to evaluate the statistical significance of nominal or dichotomous results. The Shapiro–Wilks test was used to test the normality of the distribution of continuous variables. The Mann–Whitney U-test was used to calculate the differences in non-normally distributed continuous variables. We used logistic regression to analyze the relationships between dependent dichotomous variables and independent variables. Jonckheere–Terpstra test was used to determine the trends between ordinal independent variables and continuous dependent variables. Two-sided *p* values < 0.05 were considered significant. All statistical analyses were performed using IBM’s SPSS Statistics (Armonk, NY, USA).

## Results

In total, 101 SOPPs were performed on 84 patients (14 patients had two or more SOPPs). 30 (30%) were performed at HUH, 69 (68%) at KUH, and 2 (2.0%) at SUS. The median time from the index SOPP until data analysis was 3 years (range 1–8 years). Patient and procedural characteristics are presented in Tables 1 and 2.

**Table 1:** Patient characteristics

	Mean (SD)
Age (years)	66.3 (12.5)
Body Mass Index (BMI) (kg/m^2)^	25.2 (4.21)

	*n* (%)
Sex	
Female	43 (43)
Male	58 (57)
American Society of Anesthesiologists’ physical status classification (ASA)	
ASA I	5 (5)
ASA II	49 (48)
ASA III	36 (36)
ASA IV	11 (11)

Serum carbohydrate antigen 19–9 (s-Ca19–9)	Median (range)
s-Ca19–9 (U/mL)	9.0 (< 1–865)

Maximum diameter of the pancreatic main duct according to radiology	*n* (%)
< 4.9 mm	0
5.0–9.9 mm	80 (79)
≥ 10 mm	21 (21)

**Table 2:** Procedural characteristics

	*n* (%)
Pancreatic sphincterotomy	
During SOPP	38 (38)
Earlier	53 (52)
Not done	10 (10)
Prophylactic pancreatic stent	
Yes	44 (44)
No	57 (56)
Prophylactic NSAID	
Yes	26 (26)
No	75 (74)

Procedural time	Median (range)
Minutes	74 (18–192)

*SOPP* single-operator peroral pancreatoscopy, *NSAID* nonsteroidal anti-inflammatory drug

All patients underwent MRI (*n* = 33, 33%), CT (*n* = 18, 18%), or both (*n* = 50, 50%) within 12 months prior to SOPP. Of all patients, 92 (91%) had these investigations done within 6 months prior to SOPP. EUS had been performed on 7 (7%) patients prior to SOPP. In 55 (54%) cases, there was a history of prior ERCP ± brush cytology, and among them, no malignant or HGD findings were found. In 46 (46%) cases, SOPP was the first procedure following MRI or CT imaging. The number of SOPPs during which samples were collected appears in Table 3.

**Table 3:** Combinations of samples collected during 101 SOPPs

	*n* (%)
BC + Biopsy + IF	31 (31)
BC + Biopsy	12 (12)
BC + IF	4 (4.0)
Biopsy + IF	15 (15)
BC only	4 (4.0)
Biopsy only	8 (7.9)
IF only	7 (6.9)
Visual appearance only	20 (20)

*SOPP* single-operator peroral pancreatoscopy, *BC* Brush cytology, *IF* Irrigation fluid

Ten parallel therapeutic interventions were performed at the time of SOPP including four EHL, one EHL with dilatation-assisted stone extraction (DASE), one double-balloon enteroscopy, two RFA in MD, one transduodenal SOPP, and one transduodenal Hot AXIOS™ stent placement.

### Impact on further patient care

All patients were discussed at a MDT conference following the SOPP. Of the procedures, 86 (85%) provided new information benefiting the patient and impacting further care. Indications and findings on SOPPs, and their impact on further clinical management are presented in Table 4.

**Table 4:** Indications and findings in 101 single-operator peroral pancreatoscopies, and their impact on further care

Equal possibility for pathology other than MD/MT-IPMN. SOPP indicated to exclude alternative conditions (*n* = 72, 71%)	
	Decision on further care
Tumorous tissue visible in MD suggesting MD/MT-IPMN. Alternative pathology excluded (*n* = 19)	
With HGD in BC, IF or biopsy (*n* = 2)	Surgery planned (*n* = 2)
With normal cells or LGD in IF or biopsy (*n* = 17)	Surgery planned (*n* = 12) Refused surgery, decision not based on SOPP (*n* = 5)
MD seen clear of tumorous tissue. Mucus suggests SB-IPMN (*n* = 25)	
With HGD in IF (*n* = 1)	Surgery planned (*n* = 1)
With normal cells or LGD in IF (*n* = 23)	Follow-up with MRI (*n* = 23)
With squamous cell metaplasia in biopsy (*n* = 1)	Follow-up with MRI (*n* = 1)
MD seen clear. Stricture, CP and/or pancreatic stones. No IPMN suspected (*n* = 20)	
MD clear, CP and pancreatic stones (*n* = 10)	No follow-up (asymptomatic patients) (*n* = 2) EHL performed during SOPP, no follow-up (*n* = 5) Stone removal later via ERCP (*n* = 3)
MD clear, IF and/or biopsy chronic pancreatitis (*n* = 6)	No follow-up (*n* = 6)
Post-stenting stricture seen, biopsy and/or IF samples benign (*n* = 4)	Pancreatic stent placed during SOPP, removal later No follow-up (*n* = 4)
Miscellaneous (*n* = 8)	
MD seen clear, but adenoma with LGD seen in the papilla (*n* = 1)	Endoscopic papillectomy planned (*n* = 1)
MD seen clear, but external compression from a cystadenoma suspected (*n* = 1)	Surgery planned due to cystadenomas (*n* = 1)
Visual inspection of MD unspecific, IF suggested mucus with neoplasia. Possible MD/SB-IPMN (*n* = 1)	Surgery planned (*n* = 1)
Inconclusive findings, failed to exclude MD-IPMN (*n* = 2)	Decision could not be made based on SOPP New ERCP with SOPP planned (*n* = 2)
Procedure failed, unable to enter the MD with SOPP device (*n* = 3)	Decision could not be made based on SOPP (*n* = 3)

MD/MT-IPMN likely. Risk for post-surgery complications elevated or patient is indecisive. Surgery only if SOPP demonstrates high-risk features (*n* = 24, 24%)	
	Decision on further care
Tumorous tissue visible in MD suggesting MD/MT-IPMN (*n* = 13)	
With HGD in BC, IF, or biopsy or visually massive MD-IPMN (*n* = 7)	Surgery planned (*n* = 7)
With normal cells or LGD in IF or biopsy (*n* = 6)	Close follow-up with MRI (*n* = 6)
MD seen clear. Mucus coming from a side branch or dilated openings of side branches suggesting SB-IPMN (*n* = 4)	
With HGD in IF (*n* = 1)	Surgery planned (*n* = 1)
With normal cells or LGD in IF (*n* = 2)	Follow-up with MRI (*n* = 2)
With normal cells in IF, and NET seen in papilla (*n* = 1)	Follow-up with MRI (*n* = 1)
Miscellaneous (*n* = 7)	
Post-stenting stricture seen, biopsy and IF samples benign (*n* = 6)	No follow-up planned (*n* = 6) In two of these cases SOPP failed to identify pancreatic cancer diagnosed within 4 months after SOPP
SOPP failed, unable to enter the MD with SOPP device (*n* = 1)	Decision could not be made based on SOPP (*n* = 1)

Surgery suggested. The patient is not fit for total PA. SOPP indicated to evaluate extension of IPMN lesions in MD (*n* = 5, 5.0%)	
	Decision on further care
Miscellaneous (*n* = 5)	
Tumorous tissue visible only in distal part of MD (*n* = 1)	Planned for distal pancreatic resection (*n* = 1)
Tumorous tissue visible only in proximal part of MD (*n* = 2)	Planned for pancreaticoduodenectomy (*n* = 2)
Tumorous tissue visible the entire length of the MD (*n* = 1)	Not fit for total pancreatectomy. Follow-up not continued (*n* = 1)
SOPP failed, unable to enter the MD with SOPP device (*n* = 1)	Decision could not be made based on SOPP (*n* = 1)

*MD* main pancreatic duct, *SOPP* single-operator peroral pancreatoscopy, *MD*-*IPMN* main pancreatic duct involving intraductal papillary mucinous neoplasm, *MT*-*IPMN* mixed-type intraductal papillary mucinous neoplasm, *HGD* high-grade dysplasia, *LGD* low-grade dysplasia, *SB*-*IPMN* side branch subtype of intraductal papillary mucinous neoplasm, *BC* brush cytology, *IF* irrigation fluid, *CP* chronic pancreatitis, *EHL* electrohydraulic lithotripsy, *ERCP* Endoscopic retrograde cholangiopancreatography, *NET* neuroendocrine tumor, *PA* pancreatectomy

Based on intraductal findings, surgery was planned for 29 (29%) patients. Among these patients, SOPP indicated MD-IPMN disease (*n* = 24, 24%), SB-IPMN with malignant, or HGD findings in irrigation fluid samples (*n* = 3, 3.0%) retrieved during the procedure, 1 (1.0%) adenoma of the papilla with LGD and 1 (1.0%) case of serous cystadenoma causing external compression of the MD. Among patients recommended for surgery, a more pancreatic sparing resection could be offered to 3 (3.0%) patients.

For 35 (35%) patients, follow-up with MRI was recommended based on findings in SOPP. In 29 (29%) SOPPs, the MD was seen disease-free and a SB-IPMN diagnosis was set. These patients had only benign or LGD findings in irrigation fluid samples. In four patients with SB-IPMN, SOPP discovered a coexisting condition (chronic pancreatitis *n* = 2, squamous cell metaplasia *n* = 1, and papillary neuroendocrine tumor (NET) *n* = 1). Based on SOPP findings, MDT conference recommended close follow-up with MRI to additional 6 (5.9%) patients with MD-IPMN polyps seen in MD. These patients had a high risk for surgical complications and only benign or LGD findings in biopsies and irrigation fluid samples retrieved during SOPP.

Other causes for MD dilatation were discovered in 28 (28%) patients. These included pancreatic MD stones (*n* = 10), chronic strictures caused by chronic pancreatitis or prior stenting (*n* = 16), one papillary neuroendocrine tumor (NET), and one squamous cell metaplasia. Of these patients, four were diagnosed with concurrent SB-IPMN, and for them, surveillance with MRI continued. Based on findings on SOPP, recurrent surveillance with MRI was terminated in 24 patient cases. However, of those patients, 2 (2.0%) were diagnosed with pancreatic cancer within the following 4 months after SOPP. For these two patients, SOPP was considered non-beneficial.

For 7 (6.9%) patients, the SOPP was considered as a failure and therefore not contributable to the final decision [failure to cannulate into the pancreatic MD (*n* = 5) and inconclusive findings (*n* = 2)]. Furthermore, 6 patients (5.9%) with MD-IPMN findings in SOPP refused or were unfit for surgery.

A pancreaticoduodenectomy was the most common procedure performed (*n* = 19, 66%) on patients who underwent surgery. The procedures performed are shown in Table 5. Among the 29 operated patients, visual inspection during SOPP correctly identified the intraductal pathology in 26 (90%) cases. In eight operated patients, SOPP suggested HGD or malignancy in biopsies or cytologic samples. Of these patients, 6 (75%) had HGD or malignancy in the final histopathology report. In one case, biopsies taken during SOPP failed to present HGD later discovered in the surgical specimen. The final pathological anatomic diagnosis (PAD) following surgery and their corresponding findings in SOPP appear in Table 6.

**Table 5:** Procedures performed on 29 patients undergoing surgery

Total number of patients undergoing surgery	*n* = 29 (%)
Pancreaticoduodenectomy	19 (66)
Total pancreatectomy	4 (14)
Laparotomy, found inoperable during surgery	1 (3.4)
Median pancreatectomy	1 (3.4)
Distal pancreatic resection	3 (10)
Endoscopic papillectomy	1 (3.4)

**Table 6:** Final pathological anatomic diagnosis (PAD) of 29 patients following pancreatic surgery and their corresponding findings in SOPP

Patient number	Visual and histologic or cytologic findings in SOPP	Final PAD after pancreatic surgery
1	MD/MT-IPMN, malignant	Inoperable pancreatic adenocarcinoma
2	MD/MT-IPMN, LGD	MD-IPMN, HGD, multifocal appearance of invasive growth of pancreatic adenocarcinoma
3	MD/MT-IPMN, HGD	MD-IPMN, HGD
4	MD/MT-IPMN, HGD	MD-IPMN, HGD
5	MD/MT-IPMN, HGD	MD-IPMN, HGD
6	MD/MT-IPMN, no dysplasia	MD-IPMN, LGD
7	MD/MT-IPMN, LGD	MD-IPMN, LGD
8	MD/MT-IPMN, LGD	MD-IPMN, LGD
9	MD/MT-IPMN, LGD	MD-IPMN, LGD
10	MD/MT-IPMN, LGD	MD-IPMN, LGD
11	MD/MT-IPMN, LGD	MD-IPMN, LGD
12	MD/MT-IPMN, unspecific atypia	MD-IPMN, LGD
13	MD/MT-IPMN, normal cells	MD-IPMN, LGD, autoimmune pancreatitis
14	No MD-IPMN, but HGD in IF	SB-IPMN, carcinoma in situ (PanIn3)
15	No MD-IPMN, but HGD in IF	SB-IPMN, HGD
16	No MD-IPMN, but HGD in IF	SB-IPMN, LGD
17	MD/MT-IPMN, LGD	MT-IPMN, LGD
18	MD/MT-IPMN, no specimens	MT-IPMN, HGD
19	MD/MT-IPMN, no specimens	MT-IPMN, HGD
20	MD/MT-IPMN, LGD	MT-IPMN, LGD
21	MD/MT-IPMN, LGD	MT-IPMN, LGD
22	MD/MT-IPMN, LGD	MT-IPMN, LGD
23	MD/MT-IPMN, LGD	MT-IPMN, IDP, IgG4-disease
24	MD/MT-IPMN, LGD	MT-IPMN, IDP, neuroendocrine tumor gradus 1
25	MD/MT-IPMN, normal cells	Benign serous cystadenoma
26	External compression of the MD by serous cystadenoma, no IPMN	Benign serous cystadenoma, chronic pancreatitis
27	Visual inspection unspecific, IF suggested mucus and possible IPMN with neoplasia	Chronic pancreatitis
28	MD/MT-IPMN suspected, no specimens	PanIn2, no IPMN
29	Adenoma of the papilla, LGD	LGD of the papilla, no IPMN

*SOPP* single-operator peroral pancreatoscopy, *MD*-*IPMN* main pancreatic duct involving intraductal papillary mucinous neoplasm, *HGD* high-grade dysplasia, *LGD* low-grade dysplasia, *SB*-*IPMN* side branch subtype of intraductal papillary mucinous neoplasm, *MT*-*IPMN* mixed-type intraductal papillary mucinous neoplasm, *IF* irrigation fluid, *IDP* intraductal papilloma

### Visual appearance of MD

Optical assessment of intraductal pathological findings could be obtained in 47 (47%) patients (*n* = 37 MD-IPMN, *n* = 10 pancreatic MD stones), and in another 37 (37%) patients, the MD was free from any abnormalities. Moreover, visual appearance is also a prerequisite for taking directed biopsies from pathological findings, including non-tumorous lesions such as chronic pancreatitis and iatrogenic strictures caused by previous stents. Table 7 summarizes the details for samples taken during SOPP.

**Table 7:** Samples collected during 101 SOPPs

	Brush cytology *n* (%)	Irrigation fluid *n* (%)	Biopsy *n* (%)
No sample collected	49 (49)	44 (44)	35 (35)
Inconclusive sample	1 (1.0)	13 (13)	1 (1.0)
Normal cells	29 (29)	32 (32)	26 (26)
Atypia or LGD	17 (17)	8 (7.9)	35 (35)
HGD or malignant	5 (5.0)	4 (4.0)	4 (4.0)

*SOPP* single-operator peroral pancreatoscopy, *LGD* low-grade dysplasia, *HGD* high-grade dysplasia

### Brush cytology samples

Brush cytology samples were collected during 51 (50%) SOPPs. Typically, multiple samples were taken from each patient. Brush cytology samples confirmed 5 (9.8%) cases of malignant cells and 17 (33%) cases of atypia. In the remaining samples, the finding was benign or insufficient (see Table 7 for details). Fluorescence in situ hybridization (FISH) was performed on the brush cytology samples of three (3.0%) patients. Of these three cases, one demonstrated no aneuploidy. Another one revealed aneuploidy with diploidy of the chromosomes 3, 7, 17, and the chromosome locus 9p21. FISH failed in the third case due to too few cells.

### Irrigation fluid samples

A total of 57 irrigation fluid samples were collected. Among these samples, 4 (7.0%) showed signs of a malignant or HGD disease and 11 (19%) samples indicated atypia or LGD. FISH was performed on one patient’s irrigation fluid sample. However, it failed due to insufficient number of cells. From the irrigation fluid samples, 18 (32%) samples contained mucus (see Table 7 for details).

### Biopsy samples

Visually guided biopsies (SpyBite™) were collected during 66 (67%) SOPPs. Biopsies identified 4 (6.0%) cases of HGD or malignant cells and 35 (53%) cases of atypia or LGD. In the remaining samples, the finding was benign or inconclusive. Mucin was visible in 33 (50%) samples (see Table 7).

### Post-SOPP complications

Overall, adverse events were recorded after 20 (20%) SOPPs: mild post-SOPP pancreatitis (*n* = 10, 9.9%), moderate pancreatitis (*n* = 2, 2.0%), and severe pancreatitis including one fatal outcome (*n* = 8, 7.9%). Other miscellaneous complications were 1 (1.0%) cholangitis, and 2 (2.0%) bleedings, that occurred to patients with concurrent post-SOPP pancreatitis. No patients experienced sepsis or perforation.

Among the 10 patients with parallel therapeutic interventions, five adverse events occurred to 3 (30%) patients [mild pancreatitis after RFA (*n* = 1), mild pancreatitis and bleeding after EHL + DASE (*n* = 1), and severe pancreatitis and pancreatic duct bleeding after RFA (*n* = 1)]. Of the patients who were not subjected to any additional interventions, 17 (18%) experienced a procedural associated complication. No statistically significant difference (*p* = 0.39) between the groups was found.

### Diameter of the main pancreatic duct

A Mann–Whitney U-test indicated that the median MD diameter for patients without post-SOPP pancreatitis (median = 8.0 mm) was significantly greater than for patients with post-SOPP pancreatitis (median = 6.5 mm, *p* = 0.021) (Table 8).

**Table 8:** Diameter of the main pancreatic duct and post-procedural pancreatitis. Two-tailed Mann–Whitney U-test was used with *p* values < 0.05 considered significant

	Median (mm)	25% (mm)	75% (mm)	Mann–Whitney U	*Z*-score	*p* value
Pancreatitis						
Yes (*n* = 19)	6.5	5.3	7.4	538	2.3137	0.021
No (*n* = 82)	8.0	6.0	9.0			

Analyzed as a continuous variable in logistic regression, a statistically significant decrease in odds ratio (OR) of post-SOPP pancreatitis was observed as the MD diameter increases (OR 0.714 for 1.0 mm increase in MD diameter, CI 95% 0.514–0.993; *p* = 0.045). Using the median value for MD diameter (median = 7.0 mm) as a cut-off value, logistic regression test showed a lower probability for post-SOPP pancreatitis if the MD diameter is ≥ 7.0 mm (OR 0.334, CI 95% 0.120–0.928; *p* = 0.035).

A Jonckheere–Terpstra test for ordered alternatives showed a statistically significant trend suggesting that lower median MD diameter value correlates with higher severity (scale from “mild”, “moderate” to “severe”) post-SOPP pancreatitis (*T*_JT_ = 599, SE = 116.6, *z* = − 2.31; *p* = 0.020). Table 9 summarizes median MD diameters and severity of pancreatitis.

**Table 9:** Median MD diameter values and severity of post-SOPP pancreatitis

	Median (mm)	25% (mm)	75% (mm)
No pancreatitis (*n* = 81)	7.0	6.0	9.0
Mild pancreatitis (*n* = 10)	6.3	5.8	8.1
Moderate pancreatitis (*n* = 2)	5.0	5.0	5.0
Severe pancreatitis (*n* = 8)	6.8	6.0	7.8

*MD* pancreatic main duct, *SOPP* single-operator peroral pancreatoscopy

### Prophylactic NSAID

Prophylactic NSAID (100 mg of diclofenac, rectal suppository) was used prior to endoscopy in 26 (26%) procedures. 2 (8.0%) of these patients experienced post-SOPP pancreatitis. Of the patients who had no prophylactic medication, 18 (24%) had post-SOPP pancreatitis (*p* = 0.146) (Table 10).

**Table 10:** Number of patients with post-SOPP complications and use of prophylactic NSAID, pancreatic sphincterotomy, prophylactic pancreatic stent, and history of prior pancreatitis and PEP

	No complications *n* (%)	Mild PEP *n* (%)	Moderate PEP *n* (%)	Severe PEP *n* (%)	Mild bleeding *n* (%)	Severe bleeding *n* (%)	Any complication* *n* (%)
Prophylactic NSAID							
NSAID + (*n* = 25)	23 (92)	0	0	2 (8.0)	0	0	2 (8.0)
NSAID − (*n* = 76)	58 (76)	10 (13)	2 (2.6)	6 (7.9)	1 (1.3)	1 (1.3)	18 (24)
Pancreatic sphincterotomy							
Earlier (*n* = 53)	44 (83)	6 (11)	1 (1.9)	2 (3.8)	1 (1.9)	0	9 (17)
During SOPP (*n* = 38)	28 (74)	3 (7.9)	1 (2.6)	6 (16)	0	1 (2.6)	10 (26)
Not done (*n* = 10)	9 (90)	1 (10)	0	0	0	0	1 (10)
Prophylactic pancreatic stent							
No stent (*n* = 57)	44 (77)	5 (8.8)	2 (3.5)	6 (11)	1 (1.7)	0	13 (23)
Stent (*n* = 44)	37 (84)	5 (11)	0	2 (4.5)	0	1 (2.3)	7 (16)
History of acute or chronic pancreatitis**							
Yes, acute (*n* = 17)	15 (88)	2 (12)	0	0	0	0	2 (12)
Yes, chronic (*n* = 25)	19 (76)	4 (16)	1 (4)	1 (4)	1 (4)	0	4 (24)
No acute or chronic (*n* = 62)	48 (77)	6 (9.7)	1 (1.6)	7 (11)	0	1 (1.6)	14 (23)
Papillary or MD orifice balloon dilatation							
Yes (*n* = 11)	8 (73)	2 (18)	0	1 (9.0)	0	0	3 (27)
No (*n* = 90)	73 (81)	8 (8.9)	2 (2.2)	7 (7.8)	1 (1.1)	1 (1.1)	17 (19)

	No complications *n* (%)	Mild PEP *n* (%)	Moderate PEP *n* (%)	Severe PEP *n* (%)	Mild bleeding *n* (%)	Severe bleeding *n* (%)	Any complication*** *n* (%)
History of PEP in 55 patients with prior ERCP							
Yes (*n* = 12)	7 (58)	2 (17)	1 (8.3)	2 (17)	1 (8.3)	0	5 (42)
No (*n* = 43)	39 (90)	4 (9.3)	0	0	0		4 (10)

*SOPP* single-operator peroral pancreatoscopy, *NSAID* nonsteroidal anti-inflammatory drugs, *PEP* post-ERCP pancreatitis, *ERCP* Endoscopic retrograde cholangiopancreatography, *MD* pancreatic main duct

*Please note that two patients experienced more than one complication. **Three patients had a history of both acute and chronic pancreatitis. ***One patient experienced two different complications

### Pancreatic sphincterotomy (PS)

If PS was performed alongside SOPP, pancreatitis occurred in 10 (26%) cases. Among patients, who underwent a PS at an earlier ERCP, 9 (17%) patients had post-SOPP pancreatitis (*p* = 0.306). We identified ten SOPPs performed without a PS with only 1 (10%) resulting in mild post-SOPP pancreatitis.

Among SOPPs performed on patients with a prior PS, 3 (5.6%) led to a moderate or severe pancreatitis. Meanwhile, moderate or severe pancreatitis or bleeding occurred in 7 (18%) patients who underwent PS alongside SOPP (*p* = 0.087).

### Prophylactic pancreatic stent

A prophylactic pancreatic plastic stent (10Fr) was placed during 44 (44%) of the SOPPs performed, and 7 (16%) of them led to one or more complications, while 13 (23%) SOPPs without stent placement resulted in a complication (*p* = 0.456). Moderate or severe pancreatitis or bleeding occurred in 3 (6.7%) cases among patients with a pancreatic stent and in 7 (8.2%) cases among patients without a prophylactic pancreatic stent (*p* = 0.507) (Table 10).

### History of acute or chronic pancreatitis

In 39 (39%) patient cases, there was a history of acute pancreatitis (*n* = 14, 14%), chronic pancreatitis (*n* = 22, 22%), or both (*n* = 3, 3.0%). Of the SOPPs performed on patients with prior acute pancreatitis, 2 (12%) led to mild pancreatitis, while 18 (21%) SOPPs performed on patients without prior acute pancreatitis resulted in a complication (*p* = 0.513). Of the patients with history of chronic pancreatitis, 6 (24%) suffered from post-SOPP pancreatitis. Of the complications recorded, four were graded mild and the other two were graded moderate or severe. Meanwhile, 14 (18%) of the SOPPs performed on patients without a history of chronic pancreatitis led to post-SOPP pancreatitis (*p* = 0.569) (Table 10).

### History of post-ERCP pancreatitis

One or more prior ERCPs had been performed on 55 (54%) patients. Of those 55 patients, 12 (22%) had a history of post-ERCP pancreatitis. Of these patients with prior post-ERCP pancreatitis, 5 (42%) suffered from post-SOPP pancreatitis. Meanwhile 4 (9.3%) patients with prior ERCP, but without prior complications, had post-SOPP pancreatitis (*p* = 0.017). A moderate or severe post-SOPP pancreatitis occurred 3 (25%) times to patients with history of post-ERCP pancreatitis. No cases of moderate or severe post-SOPP pancreatitis were observed in patients with prior ERCP without complications (*p* = 0.008) (Table 10).

### Papillary or MD orifice balloon dilatation

To enable entry of the SOPP device into the MD, balloon dilatation of the papillary region was performed on 11 (11%) patients. Balloons with a diameter from 4 to 6 mm were used. Of the patients with balloon dilatation, 3 (27%) experienced post-SOPP pancreatitis. Of the SOPPs performed without balloon dilatation, 17 (19%) resulted in post-SOPP pancreatitis (*p* = 0.452) (Table 10).

### Procedure time

The duration of the procedure was recorded from the beginning of the duodenoscopy through the removal of SOPP equipment from the pancreatic duct. Please note that the time includes both conventional ERCP and SOPP. The Mann–Whitney U-test revealed no statistically significant difference between procedures followed by post-SOPP complications and those with no respective complications based on the duration of the procedure (see Table 11 for details). Please note that the procedure time for six patients was unknown. Therefore, this analysis included 95 SOPPs only.

**Table 11:** Procedure duration and post single-operator peroral pancreatoscopy complications

	Median (minutes)	25% (minutes)	75% (minutes)	Mann–Whitney U	*p* value
Any complications					
No (*n* = 75)	73	50	95	617.5	0.229
Yes (*n* = 20)	82	68.5	108.5		
Pancreatitis					
No (*n* = 75)	73	50	95	617.5	0.229
Yes (*n* = 20)	82	68.5	108.5		
Moderate or severe complication					
No (*n* = 85)	73	50.5	95.5	365.0	0.474
Yes (*n* = 10)	82	63.5	107.75		

## Discussion

In 86 (85%) presumed IPMN cases, SOPP yielded additional diagnostic information benefiting the patient and impacting clinical decision-making. Previous studies have reported 64% to 77% of cholangiopancreatoscopies to carry clinical value [22, 30, 31]. The higher impact rate in this study is explained by patient selection, which differed compared to previous studies. The cohort in this study consisted not only of patients with uncertain IPMN findings, but also of patients with probable MD-IPMN, but whose comorbidities limited options for surgical treatment. We managed to demonstrate that SOPP aids clinical decision-making also in the latter patient group.

Previous studies have shown that collecting pancreatic juice with SOPP is feasible, and pancreatic juice cytology carries a diagnostic value in identifying IPMNs [28]. Studies also indicate a high specificity for detecting malignancy with biopsy specimens and irrigation fluid samples taken during SOPP [25, 27]. In this study, we identified three cases where the MD was clear from IPMN during SOPP, although irrigation fluid samples suggested IPMN disease with HGD or malignant features. In addition, visually guided biopsies taken from lesions identified in an MD and/or brush cytology samples retrieved from an MD revealed seven cases of malignancy or HGD. In this study, 75% of surgically treated patients displaying HGD or malignancy in biopsies or cytologic samples retrieved during SOPP had HGD or malignant findings in the final pathologist’s report. However, in one case, samples taken during SOPP failed to present HGD discovered later in the surgical specimen. In addition, there were two cases where SOPP failed to detect a malignancy diagnosed within 4 months of the procedure. Our study suggests that visually guided biopsies and irrigation fluid samples contribute to the diagnostic value of SOPP although risk for false negatives should be kept in mind.

The complication rate in this study was 20%. Previous studies found total complication rates of 7 to 13% for peroral cholangiopancreatoscopies [32–34]. In our study, patients underwent pancreatoscopy, and no cholangioscopies were performed. A study addressing only pancreatoscopies showed a post-SOPP pancreatitis rate of 17% [22]. Another study suggested that pancreatoscopy was associated with a higher adverse events rate of 19.8% as compared to 9.6% for cholangioscopy [31]. In this study, parallel therapeutic interventions performed during SOPPs contribute to the total complication rate (complication rate 30% versus 18% without therapeutic interventions, *p* = 0.390). Although the finding lacks statistical significance, it should be noted that all bleeding complications (*n* = 2) and 33% (*n* = 3) of all severe and moderate types of pancreatitis we observed occurred following SOPPs with parallel therapeutic interventions. Overall, the total complication rate in this study did not differ from previous studies on peroral pancreatoscopy.

A normal MD measures 1.5 to 3.5 mm in diameter [35]. In order to enter the MD, it needs to be enlarged, since the diameter of the SOPP device is approximately 3.3 mm (10 Fr) [36]. In this study, no SOPPs were performed on patients whose MD diameter fell below 5 mm. We demonstrated a decrease in odds of post-SOPP pancreatitis as the MD diameter increases (OR 0.714 for 1.0 mm increase in MD diameter, CI 95% 0.514–0.993; *p* = 0.045). A lower probability for post-SOPP pancreatitis was demonstrated, when the MD diameter is ≥ 7.0 mm (OR 0.334, CI 95% 0.120–0.928; *p* = 0.035). Furthermore, we were able to establish a trend suggesting that lower median MD diameter value correlates with higher severity post-SOPP pancreatitis (*T*_JT_ = 599, SE = 116.6, *z* = − 2.31; *p* = 0.020). Although suggested by Reuterwall et al. [31], no studies exist which address the connection between the diameter of the MD and post-SOPP pancreatitis. When planning an SOPP for a patient with MD diameter under 7.0 mm, the risk for post-SOPP pancreatitis should be carefully considered.

Multiple meta-analyses suggest that using prophylactic NSAIDs prior to conventional ERCP results in a lower post-ERCP pancreatitis rate [37–39]. Studies concerning prophylactic NSAID use specifically prior to SOPP remain scarce. In this study, patients administered with prophylactic NSAIDs experienced a lower post-SOPP pancreatitis rate compared to patients without prophylactic NSAIDs (*n* = 2 (8.0%) vs *n* = 18 (24%); *p* = 0.146). Due to a low number of patients, definitive conclusions cannot be made. However, the results agree with the recent studies concerning post-ERCP complications [37–39] and current guidelines on ERCP-related adverse events [40].

Current guidelines recommend prophylactic pancreatic stenting only in select patients at a higher risk for post-ERCP pancreatitis [40]. Furthermore, it has been suggested that prophylactic pancreatic stenting among patients with IPMN might even increase the risk for post-ERCP pancreatitis [41]. In this study, the difference in post-SOPP complication rates between patients with a prophylactic pancreatic stent versus patients without pancreatic stents was not significant (*n* = 7 (16%) vs *n* = 13 (23%); *p* = 0.456). The role of prophylactic pancreatic stenting remains controversial.

Due to the size of the SOPP device, a generous PS is typically needed in order to enter the pancreatic duct safely [42]. To our knowledge, no comprehensive studies have addressed the issue of whether there is a difference in adverse events rates, if the PS is performed simultaneously as SOPP versus previously during a different procedure. Scattered studies report individual cases of post-SOPP pancreatitis among both patients with prior PS and with PS performed simultaneously with SOPP [43, 44]. Interestingly, in this study, patients with prior PS exhibited a lower rate of moderate or severe post-SOPP pancreatitis compared with patients who underwent PS simultaneously with SOPP (*n* = 3 (5.6%) vs *n* = 7 (18%); *p* = 0.087). However, this finding was not statistically significant.

Prior pancreatitis and post-ERCP pancreatitis are known risk factors for post-ERCP pancreatitis [45]. The role of papillary balloon dilation is controversial [46]. In this study, a statistically significant difference was seen in post-SOPP pancreatitis rates between patients with prior post-ERCP pancreatitis (*n* = 5, 42%) and patients with prior ERCP without pancreatitis (*n* = 4, 9.3%, *p* = 0.017). Our results on the subject are similar to prior findings [45]. However, a statistically significant connection between post-SOPP pancreatitis and history of acute or chronic pancreatitis, or balloon dilatation of the papilla or MD orifice could not be reached.

Essentially, this first Scandinavian observational study demonstrates unambiguously the benefits of SOPP in preoperative diagnostics of suspected MD-IPMNs. However, an even larger study material should be collected to identify and confirm additional factors contributing to post-SOPP complications. In addition, we note several limitations to our study. First, the study material was collected from three different centers consisting of patients treated at different time periods. During these time periods, two different types of SOPP instrumentation were used. Second, guidelines concerning the management of MD dilatation, the use of prophylactic NSAIDs, and prophylactic stents have changed during these time periods. Furthermore, SOPPs with parallel therapeutic interventions that might contribute to the complication rate were performed in one study center only.

To conclude, patients with a dilated MD and suspected MD-IPMN lesions, but with no clear decision on subsequent care, benefit from SOPP. SOPP yields additional diagnostic information and has the potential to reduce needless surgery or unnecessary repeated surveillance with MRI within selected patients. However, the risk for post-procedural complications, especially pancreatitis is not negligible. To reduce the risk of post-SOPP complications, IPMN diagnostics with SOPP should be performed in large volume endoscopic centers with high expertise and comprehensive experience on SOPPs. Patients referred to SOPP due to indefinite IPMN findings should be discussed beforehand in a MDT conference to define the indication and expected benefit of the procedure. Patients fit for surgery with definite imaging findings supporting MD-IPMN diagnosis should be referred to surgery without performing SOPP. History of post-ERCP pancreatitis is a risk factor for post-SOPP pancreatitis. The odds for developing post-procedural pancreatitis decreases as the diameter of the MD increases. Lower values of MD diameter correlate with higher severity of post-SOPP pancreatitis. Although statistical significance was not reached here, parallel therapeutic interventions performed during SOPP might increase the risk for complications. Furthermore, the rate for moderate and severe complications might decrease when PS is performed separately from the actual SOPP, and when prophylactic NSAIDs are administered. However, these findings are not statistically significant. Finally, the use of pancreatic stents remains controversial.
